# Pregnancy Outcomes of Cervical Cerclage in a Tertiary Care Centre: A Retrospective Study

**DOI:** 10.7759/cureus.115728

**Published:** 2026-09-03

**Authors:** Deepika Sharma, Shreya Verma, Shallu Devi

**Affiliations:** 1 Obstetrics and Gynaecology, Government Medical College Jammu, Jammu, IND

**Keywords:** cervical cerclage, cervical insufficiency, neonatal, nicu, pregnancy

## Abstract

Background: Cervical insufficiency is an important cause of recurrent second-trimester pregnancy loss and spontaneous preterm delivery. Cervical cerclage is a surgery done to strengthen the cervix to prolong the pregnancy in women who need it. The result of a cerclage can vary based on the obstetric history, gestational age at the time of the cerclage, cervical length, pregnancy type, and timing of the removal.

Aim: To compare the obstetric and demographic features, timing of cervical cerclage, gestational age at delivery, and maternal and neonatal outcomes of women who underwent cervical cerclage at a tertiary-care centre.

Material and Methods: The study was a record-based observational study conducted in the Department of Obstetrics and Gynaecology, Government Medical College Jammu, in a retrospective manner. A review of the medical records was performed for 150 pregnant women who had undergone either history-indicated cervical cerclage, ultrasound-indicated cervical cerclage, or emergency cervical cerclage. A structured proforma was used to gather information about demographic data, previous obstetric history, gestational age at cerclage placement, pre-procedure cervical length, timing of cerclage removal, gestational age at delivery, birth outcome, birth weight, neonatal intensive care unit admission, and neonatal mortality. The categorical data were presented in frequencies and percentages. The Pearson chi-square goodness-of-fit test was used, and a p-value less than 0.05 was deemed to be statistically significant.

Results: The majority of participants were aged 31-40 years, accounting for 94 (62.7%) women. Most were nulliparous, 85 (56.7%), and 78 (52.0%) belonged to the middle socioeconomic group. A total of 95 (63.3%) women had experienced three or more previous abortions, while 48 (32.0%) had undergone cervical cerclage previously. Cerclage was performed at 14-17 weeks of gestation in 79 (52.7%) women, and 78 (52.0%) had a pre-procedure cervical length of more than 15 mm and up to 25 mm. Twin pregnancies accounted for 39 (26.0%) cases. Cerclage removal was performed between 36 and 37 weeks in 98 (65.3%) women. Delivery after 37 weeks occurred in 93 (62.0%) women, while 128 (85.3%) delivered at or beyond 34 weeks. The live-birth rate was 88.0% (132), NICU admission was required in 28 (18.7%) pregnancies, and neonatal mortality occurred in 6 (4.0%). Normal birth weight above 2.5 kg was recorded in 116 (77.3%) newborns. The distributions of the major study variables were statistically significant (p<0.05).

Conclusion: In most women, cervical cerclage correlated with good pregnancy and foetal outcomes. Most pregnancies were prolonged beyond 34 weeks and live-birth and neonatal survival rates were high. Proper timing of cerclage and patient selection could help to achieve better perinatal outcomes.

## Introduction

Preterm birth and mid-trimester pregnancy loss are still significant problems in obstetric care and are linked to perinatal morbidity, neonatal mortality, and long-term developmental issues in the child. These occur along different mechanisms: infection, inflammation, overdistention of the uterus and/or structural or functional weakness of the cervix. Cervical insufficiency is a condition of cervical shortening, effacement, or dilatation in the second trimester without any signs of labour. As the changes progress, the membranes may bulge or rupture, causing a miscarriage or very early preterm delivery [1]. There is no single test available for the diagnosis of cervical insufficiency, and the choice of test depends on the time of the diagnosis and the type of intervention to be undertaken. It is typically identified with a previous history of painless second-trimester losses, recurrent early preterm births, cervical shortening seen on transvaginal ultrasonography, or painless cervical dilatation on examination. This is a complex process that can be caused by changes in the organization of collagen, a decrease in the tensile strength of the cervix, abnormal remodelling of the extracellular matrix, congenital defects, or acquired damage [2]. Cervical integrity may be compromised by previous cervical surgery, repeated mechanical dilatation, traumatic delivery, cervical laceration, or conization.

Measurement of cervical length by transvaginal ultrasound is a key component of the evaluation of women at risk for spontaneous preterm birth, but many women affected do not have a clearly identifiable structural defect. It detects a reduction in cervical length before it becomes apparent, and allows repeat measurements if any progressive change is suspected. The value of cervical length should be used in conjunction with the gestational age, type of pregnancy, previous obstetric history, symptoms, and cervical dilatation. Having a short cervix does not mean she has an insufficiency problem; not all women with a short cervix will need surgery [3].

The current recommendations are to use cervical cerclage, a surgical technique that involves tying a non-absorbable suture around the cervix to strengthen support and prevent further cervical opening. It can be either history-indicated, ultrasound-indicated, or physical examination-indicated. Cerclage is scheduled in women who have had a previous loss or early labor, and for whom a history would suggest this. When cervical shortening occurs in a woman who has relevant obstetric risk factors, ultrasound-indicated cerclage is considered an option. Physical examination-indicated, emergency, or rescue cerclage is done when painless cervical dilatation has already occurred, possibly accompanied by exposed fetal membranes [4]. The principal transvaginal techniques are the McDonald technique and the Shirodkar technique, which should be considered prior to placement due to the factors of gestational age and membrane status, and because there are no reports of labour or infection associated with these techniques. The McDonald method is relatively easy to perform and remove, whereas the suture position is more internal with the Shirodkar method, and involves more dissection.

Preoperative evaluation involves confirming viability of the fetus, checking for contractions, bleeding, rupture of membranes, fetal anomaly, and infection. Patient selection is critical with emergency cerclage due to potential risks of membrane rupture, ascending infection, and technical difficulty with advanced dilatation of the cervix and exposed fetal membranes. In selected women, where a cerclage can not be performed in the vagina, or where it has already been performed but failed, the choice should be made taking into account the potential risks and benefits of the two procedures. It can be performed before pregnancy or early pregnancy (Open or Laparoscopic) [5]. This is only used for high-risk patients and where delivery is only possible by caesarean section as the suture stays at the cervico-isthmic level. Postcerclage follow-up should focus on symptoms of preterm labor, ruptured membranes, infection, cervical trauma and fetal well-being. Transvaginal cerclage is usually removed at about 37 weeks in uncomplicated cases in which vaginal birth is intended, but earlier removal may be necessary if labour occurs, if there is bleeding or infection, or if rupture of membranes occurs [6]. So, the present study was undertaken to compare the obstetric and demographic features, timing of cervical cerclage, gestational age at delivery, and maternal and neonatal outcomes of women who underwent cervical cerclage at a tertiary-care centre.

## Materials and methods

Study design and setting

This retrospective, record-based observational study was conducted in the Department of Obstetrics and Gynaecology, Government Medical College and Hospital, Jammu, a tertiary-care referral centre providing specialized obstetric and neonatal services. The study evaluated maternal and neonatal outcomes among pregnant women who underwent cervical cerclage at the institution between January 1, 2025, and December 31, 2025. Data were obtained retrospectively from routinely maintained hospital records; therefore, there was no prospective recruitment or direct interaction with participants for this study.

Ethical approval

The research protocol was reviewed by the Institutional Ethics Committee, Government Medical College, Jammu, Sub-Committee, and was “Approved Provisionally” as per the approval issued vide No. IEC/GMCJ/2026/2769, dated 7 March 2026. The project was classified under Category C (Cat-C) according to the applicable Standard Operating Procedures and Indian Council of Medical Research guidelines and was registered under Registration No. C-681.

Study population

The source population comprised pregnant women managed in the Department of Obstetrics and Gynaecology during the study period. The study population consisted specifically of women who underwent cervical cerclage between January 1, 2025, and December 31, 2025, and whose medical records contained sufficient information to determine the indication for cerclage, relevant maternal and obstetric characteristics, and pregnancy and neonatal outcomes. Patients were followed through the available hospital records till March 31, 2026, or until delivery or pregnancy loss. Patients for whom pregnancy outcome was not available at the time of data collection were excluded from outcome analysis. We had not included patients whose pregnancy outcome was not documented in the hospital records by March 31, 2026. Women undergoing history-indicated, ultrasound-indicated, or emergency/rescue cervical cerclage were eligible. Both singleton and multifetal pregnancies were considered.

For the purpose of this study, history-indicated cerclage referred to cerclage performed on the basis of a relevant previous obstetric history suggestive of cervical insufficiency. Ultrasound-indicated cerclage referred to cerclage performed following sonographic evidence of cervical shortening in a woman considered to be at increased risk of preterm birth. Emergency/rescue cerclage referred to cerclage performed when cervical dilatation was detected during pregnancy in the absence of active labour, major vaginal bleeding, or clinical evidence of intra-amniotic infection/chorioamnionitis.

Sampling technique

A non-probability, consecutive sampling technique with complete enumeration was used. All eligible medical records of women who underwent cervical cerclage during the study period were screened. Records fulfilling the eligibility criteria were included consecutively until the required sample size was achieved. A total of 150 patients were included in the final analysis.

Sample size calculation

The minimum sample size was estimated using the single-population proportion formula:

\begin{equation} n = \frac{Z^{2} \, p(1-p)}{d^{2}} \end{equation}

Assuming a 95% confidence level, an anticipated proportion of 50% (p = 0.50), and an absolute precision of 8%, the minimum required sample size was calculated using the formula (n = Z^2p(1-p)/d^2). Accordingly, (n = (1.96)^2 \times 0.50 \times 0.50/(0.08)^2 = 150.06). Thus, the minimum required sample size was approximately 150 participants. All 150 eligible records with adequate information were included in the study.

Inclusion criteria

Pregnant women were included in the study if they underwent cervical cerclage during the specified study period at Government Medical College and Hospital Jammu. Eligible women were those with either singleton or multifetal pregnancies. Patients were included who had a history of indicated, ultrasound-indicated, and emergency/rescue cervical cerclages. Only women with full medical records of length of gestation at time of cerclage placement, cervical findings, obstetric history, timing of cerclage removal, period of gestation at delivery, and pregnancy outcome were included.

Exclusion criteria

Records were excluded if the pregnancy outcome could not be determined from the available documentation or if essential clinical information was missing. Women with major fetal congenital or chromosomal abnormalities diagnosed before cerclage placement and women with intrauterine fetal death before cerclage placement were excluded. Women with active labour, severe vaginal bleeding, or clinical chorioamnionitis at the time of assessment for cerclage were excluded. Records relating to previous cerclage procedures without adequate procedural or follow-up documentation were not considered for analysis of those previous procedures. Pregnancies terminated for maternal or fetal indications unrelated to cervical insufficiency were also excluded.

Methodology

Data were collected retrospectively from antenatal records, inpatient case files, operation theatre registers, labour room registers, discharge records, neonatal records, and available follow-up records. Information was extracted systematically using a structured data-collection proforma to maintain uniformity across records. Demographic and maternal variables included maternal age, parity, educational status, socioeconomic status, and body mass index (BMI). Previous obstetric and cervical history included previous abortions, previous live births, history of spontaneous preterm birth, previous cervical cerclage, number and outcomes of previous cerclage procedures, previous traumatic cervical laceration, and history of loop electrosurgical excision procedure (LEEP) or cervical conization. Current pregnancy variables included mode of conception, in vitro fertilization (IVF) conception, number of fetuses, gestational age at cerclage placement, cervical length before cerclage, indication for cervical cerclage, and timing of cerclage removal.

Outcome assessment and operational definitions

Pregnancy and neonatal outcomes were determined retrospectively from labour room records, inpatient case records, delivery records, discharge summaries, and neonatal records. Gestational age at delivery and neonatal status documented at birth and during the neonatal period were used for outcome classification. The primary pregnancy outcomes were gestational age at delivery and live birth. Gestational age at delivery was categorized as <28 weeks, 28 to <34 weeks, 34 to <37 weeks, and ≥37 weeks. For regression analyses examining term delivery, a favourable outcome was defined as delivery at or beyond 37 completed weeks of gestation.

Live birth was defined as delivery of a fetus showing evidence of life after complete expulsion or extraction from the mother, irrespective of gestational age. Stillbirth was defined according to the definition adopted in the hospital records applicable during the study period. Cases were classified as stillbirth when the delivery record documented fetal death at birth meeting the institutionally applied gestational-age/birth-weight criteria. NICU admission was defined as admission of a live-born neonate to the neonatal intensive care unit following delivery, as documented in the neonatal or hospital record. The variable was recorded dichotomously as admitted or not admitted. Neonatal mortality was defined as death of a live-born infant within the first 28 completed days after birth, where this information was available in the neonatal or follow-up record. Birth weight was based on the weight documented in the neonatal/delivery record and was categorized for study analysis as very low birth weight (<1.5 kg), low birth weight (1.5-2.5 kg), and normal birth weight (>2.5 kg). Secondary outcomes therefore included timing of cerclage removal, live birth, stillbirth, birth weight, NICU admission, and neonatal mortality.

Potential confounding factors

Potential confounding variables were identified a priori on the basis of their clinical relevance to pregnancy and neonatal outcomes. These included maternal age, parity, BMI, socioeconomic status, educational status, history of previous abortions, previous spontaneous preterm birth, previous live birth, previous cervical cerclage and its outcome, cervical length before cerclage placement, indication for cerclage, previous cervical trauma, previous loop electrosurgical excision procedure (LEEP) or conization, in vitro fertilization (IVF) conception, and singleton versus multifetal pregnancy.

Potential confounding was addressed through stratified analyses and, where the number of outcome events permitted reliable estimation, multivariable binary logistic regression. Variables considered clinically important and variables showing an association at p<0.20 in univariable analysis were considered for inclusion in the multivariable model. Adjusted odds ratios (aORs) with 95% confidence intervals (CIs) were calculated to estimate the independent association of the included factors with favourable pregnancy outcomes. Multicollinearity and overall model fit were assessed before interpretation of the final model.

Statistical analysis

The collected data were checked for completeness and consistency before analysis. Categorical variables were summarized using frequencies and percentages, n (%). Demographic characteristics, previous obstetric and cervical history, current pregnancy and cerclage characteristics, timing of cerclage removal, gestational age at delivery, and pregnancy and neonatal outcomes were primarily analyzed descriptively. A chi-square test was used to determine if there is a significant difference between observed and expected frequencies in categorical data.

Where associations between clinically relevant categorical variables and pregnancy outcomes were evaluated, Pearson's chi-square test of independence or Fisher's exact test, as appropriate, was used. Multivariable binary logistic regression was performed where the number of outcome events permitted reliable estimation to identify factors independently associated with favourable pregnancy outcomes. Effect estimates are reported as odds ratios with 95% confidence intervals. A two-sided p-value <0.05 was considered statistically significant for inferential analyses.

## Results

Demographic characteristics

The demographic data of the women managed with cervical cerclage are summarised in Table 1. Most of the participants were in the age group 31 to 40 years (94 women, 62.7%), and 48 women (32.0%) were aged 21 to 30 years, with only 8 women (5.3%) aged 41 to 50 years. The age distribution was found to be statistically significant (χ²=74.08, DF=2, p<0.001), meaning that women aged 31 to 40 had the highest number of cervical cerclage (Table 1).

**Table 1:** Demographic characteristics of patients managed with cervical cerclage (N=150) Chi-square test was applied to compare the variables. p<0.05 was considered significant

Characteristic	Category	n (%)	χ² (df)	p-value
Age (years)	21–30	48 (32.0%)	74.08 (2)	<0.001
	31–40	94 (62.7%)		
	41–50	8 (5.3%)		
Parity	0	85 (56.7%)	40.36 (2)	<0.001
	1	42 (28.0%)		
	≥2	23 (15.3%)		
Educational status	None	4 (2.7%)	77.73 (3)	<0.001
	Primary	26 (17.3%)		
	Secondary	42 (28.0%)		
	Tertiary	78 (52.0%)		
Socioeconomic status	Lower	43 (28.7%)	25.48 (2)	<0.001
	Middle	78 (52.0%)		
	Upper	29 (19.3%)		
BMI (kg/m²)	<18.5	12 (8.0%)	59.01 (3)	<0.001
	18.5–24.9	67 (44.7%)		
	25.0–29.9	54 (36.0%)		
	≥30	17 (11.3%)		

In terms of parity, the number of women with no previous delivery was 85 (56.7%) while the number of women with one previous delivery was 42 (28.0%) and those who had experienced two or more previous deliveries were 23 (15.3%). There was a significant difference in parity category distribution (χ²=40.36, df=2, p<0.001), with over 50% of women who had a cervical cerclage not having had a viable childbirth previously. In terms of education level, 78 (52.0%) women had tertiary education, 42 (28.0%) secondary education, 26 (17.3%) primary education, and 4 (2.7%) had no formal education. The educational distribution showed a significant difference (χ²=77.73, df=3, p<0.001), indicating that higher educational levels were obtained by the majority of the women who seek care at the tertiary centre.

The socioeconomic profile revealed that 28.7% (43) of the women were from the lower socioeconomic class, 19.3% (29) of the women were from the upper socioeconomic class, and 52.0% (78) of the women were from the middle socioeconomic class. The distribution was statistically significant (χ²=25.48, df=2, p<0.001) and the largest group of the study population was the middle socioeconomic group. Assessment of body mass index revealed that 67 (44.7%) women had a normal BMI (18.5-24.9 kg/m²), 54 (36.0%) were overweight (25.0-29.9 kg/m²), 17 (11.3%) were obese (≥30 kg/m²), and 12 (8.0%) were underweight (<18.5 kg/m²). There was a significant difference in the BMI distribution between categories (χ²=59.01, df=3, p<0.001). Overall, more than four-fifths of the women had either normal body weight or were overweight (Table 1).

Obstetrics and cervical history

The participants' previous obstetric and cervical history is shown in Table 2. Recurrent pregnancy loss was also frequent, as 63.3% (95) women had three or more previous abortions, while 22.7% (34) had experienced two abortions and 14% (21) had experienced one abortion. The distribution of indications observed was statistically significant (χ²=62.44, df=2, p<0.001), and showed that recurrent miscarriage was an important indication for cervical cerclage. A total of 54 women (36.0%) had experienced a previous live birth and 96 women (64.0%) had never had a live birth. This difference was statistically significant (χ²=11.76, df=1, p<0.001), with almost two-thirds of subjects never achieving a viable pregnancy before.

Most women did not have a previous traumatic laceration of the cervical area, while 4 (2.7%) did. The distribution was very significant (χ²=134.43, df=1, p<0.001), showing that cervical trauma after childbirth was a rare antecedent among women who had a cerclage. Likewise, 18 (12.0%) females had a prior history of spontaneous preterm delivery, whilst 132 (88.0%) had no documented history of preterm delivery. This difference was found to be statistically significant as per the chi-squared value obtained (χ²=86.64, df=1, p<0.001). So, for most women, the indication for cervical cerclage was due to other risk factors, such as recurrent pregnancy loss or cervical shortening.

In 48 (32.0%) women, a previous cerclage had been performed; in 102 (68.0%) women, the cerclage was done for the first time. This distribution was statistically significant (χ²=19.44, df=1, p<0.001). The difference in the number of previous cerclages between the 48 women with a previous cerclage (35 women performed one cerclage, 13 women performed more than one) was statistically significant (χ²=10.08, df=1, p=0.001). Moreover, of the women who had a previous pregnancy, 34 (70.8%) had a favourable outcome while 14 (29.2%) had failed a previous cerclage (χ²=8.33, df=1, p=0.004), indicating previous cervical cerclage had been successful in most of these women (Table 2).

**Table 2:** Previous obstetric and cervical history of the study participants (N=150) Chi-square test was applied to compare the variables. p<0.05 was considered significant

Characteristic	Category	n (%)	χ² (df)	p-value
Number of prior abortions	1	21 (14.0%)	62.44 (2)	<0.001
	2	34 (22.7%)		
	≥3†	95 (63.3%)		
Prior live birth	Yes	54 (36.0%)	11.76 (1)	<0.001
	No	96 (64.0%)		
Prior cervical laceration following traumatic birth	Yes	4 (2.7%)	134.43 (1)	<0.001
	No	146 (97.3%)		
History of preterm birth	Yes	18 (12.0%)	86.64 (1)	<0.001
	No	132 (88.0%)		
Past history of cervical cerclage	Yes	48 (32.0%)	19.44 (1)	<0.001
	No	102 (68.0%)		
Number of previous cerclages	1	35 (72.9%)	10.08 (1)	0.001
	>1	13 (27.1%)		
Outcome of previous cerclage	Favourable	34 (70.8%)	8.33 (1)	0.004
	Previous failed cerclage	14 (29.2%)		

Current pregnancy and cervical cerclage characteristics

Characteristics of the current pregnancy and cervical cerclage procedure are described in Table 3. The 14-17 week period was the most common time for cerclage, with 79 (52.7%) women having it performed during this time. Twenty (13.3%) had cerclage performed between 20 and 24 weeks, and another 51 (34.0%) had it performed between 17 and 20 weeks. Mean gestational age at cerclage placement was statistically different (χ²=34.84, df=2, p<0.001), with the majority of patients in the early second trimester. In addition, 78 (52 %) women had a cervical length of more than 15 mm but less than 25 mm and 54 (36.0%) had a cervical length of 10 mm to 15 mm, while 18 (12.0%) had a cervical length of less than 10 mm prior to the placement of cerclage. The distribution was statistically significant (χ²=36.48, df=2, p<0.001). So, more than half of the women had a cerclage performed prior to severe cervical shortening.

Singleton pregnancies were most common, with 111 (74.0%) pregnancies identified, and 39 (26.0%) pregnancies identified as twin pregnancies. There were no multiple pregnancies of three or more. This distribution was statistically significant (χ²=126.84, df=2, p<0.001), which showed that the majority of patients had a singleton pregnancy during the time of the cerclage procedure. In vitro fertilization was the cause of only 25 pregnancies (16.7%), while 125 pregnancies (83.3%) were conceived naturally. The distribution was statistically significant (χ²=66.67, df=1, p<0.001), and showed that spontaneous conception was the dominant mode of conception in the study population. Only one woman (0.7%) had a prior history of cervical LEEP or conization; 149 (99.3%) did not have a history of cervical excisional procedures. This difference was highly significant (χ²=146.03, df=1, p<0.001), indicating that previous cervical surgery was a minor contributor in this cohort (Table 3).

**Table 3:** Current pregnancy and cervical cerclage characteristics (N=150) Chi-square test was applied to compare the variables. p<0.05 was considered significant

Characteristic	Category	n (%)	χ² (df)	p-value
Gestational age at cerclage placement	14–17 weeks	79 (52.7%)	34.84 (2)	<0.001
	>17–20 weeks	51 (34.0%)		
	>20–24 weeks	20 (13.3%)		
Cervical length before the procedure	<10 mm	18 (12.0%)	36.48 (2)	<0.001
	10–15 mm	54 (36.0%)		
	>15–25 mm	78 (52.0%)		
Number of fetuses	Singleton	111 (74.0%)	126.84 (2)	<0.001
	Twins	39 (26.0%)		
	≥3	0 (0.0%)		
IVF conception	Yes	25 (16.7%)	66.67 (1)	<0.001
	No	125 (83.3%)		
Previous LEEP/conization	Yes	1 (0.7%)	146.03 (1)	<0.001
	No	149 (99.3%)		

Timing of cerclage removal and gestational age at delivery

Table 4 shows the time of cerclage removal and gestational age at delivery. Cerclage removal was performed between 36 and 37 weeks of gestation in 98 (65.3%) women, before 36 weeks in 37 (24.7%), and after 37 weeks in 15 (10.0%). This distribution was statistically significant (χ²=73.96, df=2, p<0.001) and showed that elective removal was most common at 36-37 weeks, indicating the normal time period for most pregnancies. In terms of gestational age at birth, 93 (62.0%) women delivered after 37 completed weeks; 35 (23.3%) had deliveries between 34 and 37 weeks. Between 28 and less than 34 weeks, deliveries were made in 18 women (12.0%), while 4 (2.7%) delivered before the age of 28 weeks. The results of the distribution were statistically significant (χ²=122.37, df=3 and p<0.001). 

**Table 4:** Timing of cerclage removal and gestational age at delivery (N=150) Chi-square test was applied to compare the variables. p<0.05 was considered significant

Characteristic	Category	n (%)	χ² (df)	p-value
Timing of cerclage removal	<36 weeks	37 (24.7%)	73.96 (2)	<0.001
	36–37 weeks	98 (65.3%)		
	>37 weeks	15 (10.0%)		
Gestational age at delivery	<28 weeks	4 (2.7%)	122.37 (3)	<0.001
	28–<34 weeks	18 (12.0%)		
	34–37 weeks	35 (23.3%)		
	>37 weeks	93 (62.0%)		

Pregnancy outcomes and outcomes in babies

Table 5 shows the birth and neonatal outcomes in pregnancies after cervical cerclage. Of 132 pregnancies, 88.0% gave birth to a live baby and (18) 12.0% were stillborn. This difference was statistically significant (χ²=86.64, df=1, p<0.001), and showed that overall live-birth rate following cervical cerclage was high. Of the 28 pregnancies admitted to the neonatal intensive care unit, 18.7% required NICU care, and 122, 81.3% did not require NICU care. This distribution was statistically significant (χ²=58.91, df=1, p<0.001), and most neonates were sufficiently well after birth that they did not require intensive neonatal care.

**Table 5:** Pregnancy-level birth and neonatal outcomes following cervical cerclage (N=150 pregnancies) Chi-square test was applied to compare the variables. p<0.05 was considered significant

Outcome	Category	n (%)	χ² (df)	p-value
Pregnancy birth outcome	Live birth	132 (88.0%)	86.64 (1)	<0.001
	Stillbirth	18 (12.0%)		
Pregnancies requiring NICU admission§	Yes	28 (18.7%)	58.91 (1)	<0.001
	No	122 (81.3%)		
Pregnancies with neonatal mortality§	Yes	6 (4.0%)	126.96 (1)	<0.001
	No	144 (96.0%)		
Birth-weight category¶	Very low birth weight, <1.5 kg	9 (6.0%)	133.24 (2)	<0.001
	Low birth weight, 1.5–2.5 kg	25 (16.7%)		
	Normal birth weight, >2.5 kg	116 (77.3%)		

Neonatal mortality was seen in 6 (4.0%) pregnancies and neonatal survival in 144 (96.0%) pregnancies. The difference was statistically significant (χ²=126.96, df=1, p<0.001) and showed very good neonatal survival rates in the study population. Regarding birth weight, there were 116 (77.3%) pregnancies in which a newborn weighed over 2.5 kg, 25 (16.7%) in which a newborn weighed between 1.5 and 2.5 kg and 9 (6.0%) in which a newborn weighed under 1.5 kg. The distribution of birth weight categories was statistically significant (χ²=133.24, df=2, p<0.001).

## Discussion

In the present study, 150 women undergoing cervical cerclage were included, of whom 94 (62.7%) were aged 31-40 years and 48 (32.0%) were aged 21-30 years. The parity distribution included 85 (56.7%) nulliparous women, 42 (28.0%) women with parity one, and 23 (15.3%) women with parity two or more. Furthermore, 78 (52.0%) women had received tertiary education, 78 (52.0%) belonged to the middle socioeconomic group, 67 (44.7%) had a normal BMI, 54 (36.0%) were overweight, and 17 (11.3%) were obese. In the study by Ikechebelu et al. (2023), among 103 women undergoing cerclage due to a history of or ultrasound evidence of cervical insufficiency, 54.4% were aged between 24 and 34 years, while 37.9% were aged between 35 and 45 years [7]. There were no significant sociodemographic differences among women who underwent a history-indicated or ultrasound-indicated procedure. The comparatively higher percentage of women aged 31-40 years in the present study could be attributed to delayed pregnancy, recurrent pregnancy loss, infertility treatment, and referral of older high-risk pregnant women to a tertiary-care centre. The fact that the majority of women in the current study were nulliparous further suggests that they were selected primarily because of previous pregnancy losses rather than previous live births [7].

A significant proportion of women had previous adverse pregnancy outcomes, as 95 (63.3%) had experienced three or more abortions and 96 (64.0%) had no previous live birth. Only 18 (12.0%) had a history of preterm birth; however, many previous adverse outcomes may have occurred as mid-trimester pregnancy losses rather than viable preterm births. Forty-eight women (32.0%) reported a previous cervical cerclage. Among these women, 35 (72.9%) had undergone one previous cerclage, while 34 (70.8%) had experienced a favourable outcome following the previous procedure. In a retrospective cohort of 399 singleton pregnancies in women who underwent history-indicated cerclage, Lee et al. (2018) [8] found that 15 of 194 women (8%) with one previous second-trimester loss and 28 of 205 women (14%) with two or more previous losses delivered before 32 weeks. Women with one previous loss also had a lower NICU admission rate than those with two or more losses, at 10% and 17%, respectively. These observations further suggest that the number of previous pregnancy losses is important for identifying women at higher risk of recurrent early delivery, even after cerclage [8].

A history of previous traumatic cervical laceration was uncommon in the present study and was reported in only 4 (2.7%) women, whereas 146 (97.3%) had no such history. Likewise, only one woman (0.7%) had undergone a previous LEEP or cervical conization, suggesting that most cases of suspected cervical insufficiency were not associated with previous traumatic or surgically acquired cervical damage. Wong et al. (2016) studied 55 women who had experienced a cervical laceration requiring repair during a previous delivery, and two of these women (4.6%) underwent cerclage in the subsequent pregnancy. One woman delivered at term, while the other delivered at 35 weeks and 6 days. Overall, only four women (9.3%) delivered before 37 weeks in the subsequent pregnancy, and one woman experienced recurrent cervical laceration. These findings are consistent with the low incidence of cervical trauma observed in the present study and suggest that previous cervical laceration is an uncommon indication for cerclage [9].

In the present study, 79 (52.7%) women underwent cerclage at 14-17 weeks of gestation, 51 (34.0%) underwent the procedure after 17 weeks and up to 20 weeks, and 20 (13.3%) underwent cerclage after 20 weeks and up to 24 weeks. Before the procedure, 78 (52.0%) women had a cervical length of more than 15 mm and up to 25 mm, 54 (36.0%) had a cervical length of 10-15 mm, and 18 (12.0%) had a cervical length below 10 mm. Golbasi et al. (2022) reported that the mean gestational ages at cerclage placement for history-indicated, ultrasound-indicated, and physical examination-indicated cerclage were 16.1±2.7, 18.1±3.5, and 20.6±1.5 weeks, respectively [10]. The corresponding mean cervical lengths were 27.3±6.9 mm, 17.1±3.4 mm, and 17.2±6.3 mm. Deliveries before 28 weeks occurred in 9.8%, 5.9%, and 33.3% of these groups, while deliveries before 34 weeks occurred in 26.8%, 11.8%, and 60%, respectively. With a predominance of cerclage placement before 20 weeks in the current study, the relatively low rates of extremely and very preterm delivery may have been partly related to earlier intervention [10]. However, the present study does not establish a causal relationship between the timing of cerclage and pregnancy prolongation.

In the current study, 111 (74.0%) pregnancies were singleton pregnancies and 39 (26.0%) were twin pregnancies, with no cases involving three or more fetuses. Twenty-five women (16.7%) conceived through IVF, while 125 (83.3%) conceived without IVF. Kumari et al. (2023) compared 129 women, including 81 who underwent elective cerclage and 48 who underwent emergency cerclage, and found that the frequency of twin pregnancy was almost four times higher in the emergency cerclage group than in the elective cerclage group. The mean cervical length at cerclage was 2.05 cm in the elective group and 1.5 cm in the emergency group. Delivery at or beyond 34 weeks occurred in 71% of the elective group and 53.3% of the emergency group, while live-birth rates were 84.4% and 81.3%, respectively. Composite neonatal morbidity was higher following emergency cerclage than elective cerclage, at 35.5% versus 14%. The inclusion of 26.0% twin pregnancies in the present study is clinically relevant because multifetal gestation is associated with increased risks of cervical shortening, preterm delivery, low birth weight, and neonatal morbidity [11].

Cerclage was removed between 36 and 37 weeks in 98 (65.3%) women, before 36 weeks in 37 (24.7%), and after 37 weeks in 15 (10.0%). Ninety-three women (62.0%) delivered after 37 weeks, 35 (23.3%) delivered between 34 and 37 weeks, 18 (12.0%) delivered between 28 and less than 34 weeks, and only 4 (2.7%) delivered before 28 weeks. Overall, 128 (85.3%) women delivered at or beyond 34 weeks of gestation. Chan et al. (2015) analyzed 47 singleton pregnancies and found that 28 women (59.6%) delivered after 34 weeks, with a median gestational age at delivery of 35.7 weeks [12]. The median gestational age at delivery following history-indicated cerclage was 37.6 weeks, compared with 35.4 weeks following ultrasound-indicated cerclage and 24.3 weeks following rescue cerclage. Delivery before 34 weeks occurred in 26.1%, 40.0%, and 77.8% of the respective groups. The rate of delivery at or beyond 34 weeks in the present study was 85.3%, which was higher than the 59.6% reported by Chan et al. This difference may be explained by the fact that a substantial proportion of women in the present study underwent cerclage during the early second trimester before the development of advanced cervical dilatation or prolapsed membranes [12].

In the present study, 132 pregnancies resulted in live birth and 18 resulted in stillbirth, corresponding to a live-birth rate of 88.0%. NICU admission was required in 28 (18.7%) pregnancies, while neonatal mortality occurred in 6 (4.0%), resulting in an overall neonatal survival rate of 96.0%. Pang et al. (2019) studied 50 women with singleton pregnancies who underwent emergency cerclage for cervical insufficiency with prolapsed membranes [13]. The mean gestational age at cerclage was 21.3±2.2 weeks, the mean interval between cerclage and delivery was 11.2±7.1 weeks, and the mean gestational age at delivery was 34.1 weeks. Twenty neonates, representing 40% of the study population, required NICU admission, and only 10 pregnancies continued beyond 36 weeks. The lower NICU admission rate of 18.7% in the current study may be related to the higher proportion of women undergoing planned cerclage before the development of advanced cervical changes and the greater proportion of pregnancies continuing beyond 37 weeks [13].

Regarding birth weight, 116 (77.3%) pregnancies resulted in newborns weighing more than 2.5 kg, 25 (16.7%) had low-birth-weight newborns weighing between 1.5 and 2.5 kg, and 9 (6.0%) had very-low-birth-weight newborns weighing less than 1.5 kg. Thus, 34 (22.7%) pregnancies resulted in newborns with either low or very low birth weight. In a study of emergency cerclage using a three-step procedure, Gómez-Castellano et al. (2022) reported a mean gestational age at delivery of 35 weeks, a mean neonatal birth weight of 2550 g, and a neonatal survival rate of 95.8% [14]. Premature rupture of membranes before 34 weeks occurred in 8.3% of cases. Their neonatal survival rate was almost identical to the 96.0% recorded in the present study, while the finding that 77.3% of newborns in the present study weighed more than 2.5 kg suggests generally favourable fetal growth and prolongation of pregnancy. However, because of the retrospective nature of the present study and the absence of an untreated control group, the favourable findings should be interpreted as outcomes observed following cerclage rather than as definitive effects directly caused by cerclage [14].

Limitations

There were some limitations to this study, including its retrospective, single-centre design that may limit the generalizability of the results. Medical records might have been incomplete and documentation bias may have occurred due to reliance on them. An untreated control group was not available for direct evaluation of the effectiveness of cervical cerclage. Furthermore, the associations observed might have been confounded due to the small sample size.

## Conclusions

In the majority of women in this study with cervical cerclage, pregnancy and neonatal outcomes were good. Most of the infants were born at or after 34 weeks of gestation, 62% of whom went beyond 37 weeks. High live-birth rate, low neonatal mortality with predominant normal birth weight were observed. The results corroborate the use of timely and properly placed cervical cerclage to extend pregnancy and enhance perinatal outcomes.
